# Alkylamidobetaines Induced Changes in Multidrug-Resistant, Biofilming *Candida* *glabrata*—Analysis of Oxidative Stress and Cellular Proteome

**DOI:** 10.3390/ijms27188309

**Published:** 2026-09-18

**Authors:** Mariusz A. Bromke, Emilia Nowak, Olga Bortkiewicz, Bartosz Poniewierka, Michał Gleńsk, Urszula Nawrot, Marcin Luzarowski, Jarosław Widelski, Łukasz Lamch, Kazimiera A. Wilk, Emil Paluch

**Affiliations:** 1 Department of Biochemistry and Immunochemistry, Wroclaw Medical University, Ul. Chałubińskiego 10, 50-368 Wroclaw, Poland; mariusz.bromke@umw.edu.pl; 2 Department of Microbiology, Faculty of Medicine, Wroclaw Medical University, Ul. Chałubińskiego 4, 50-376 Wroclaw, Poland; apulapuczija@gmail.com (E.N.); olga.bortkiewicz@umw.edu.pl (O.B.); 3 Faculty of Medicine, SKN Medical Image Analysis and Research in Pathology (MIARP), Wroclaw Medical University, Wyb. Pasteura 1, 50-375 Wroclaw, Poland; bartosz.poniewierka@student.umw.edu.pl; 4 Department of Pharmacognosy and Herbal Medicines, Wroclaw Medical University, Ul. Borowska 211a, 50-556 Wrocław, Poland; michal.glensk@umw.edu.pl; 5 Department of Pharmaceutical Microbiology and Parasitology, Wroclaw Medical University, Ul. Borowska 211a, 50-556 Wrocław, Poland; urszula.nawrot@umw.edu.pl; 6 Core Facility for Mass Spectrometry and Proteomics, Center for Molecular Biology of Heidelberg University (ZMBH), DKFZ-ZMBH Alliance, Im Neuenheimer Feld 329, 69120 Heidelberg, Germany; m.luzarowski@zmbh.uni-heidelberg.de; 7 Department of Pharmacognosy with Medicinal Plants Garden, Lublin Medical University, Ul. Chodźki 1, 20-093 Lublin, Poland; jaroslaw.widelski@umlub.edu.pl; 8 Department of Engineering and Technology of Chemical Processes, Wroclaw University of Science and Technology, Norwida 4/6, 50-373 Wroclaw, Poland; lukasz.lamch@pwr.edu.pl (Ł.L.); kazimiera.wilk@pwr.edu.pl (K.A.W.)

**Keywords:** *Candida glabrata*, surfactants, oxidative stress, alkylamidobetaines, proteomics

## Abstract

Antibiofilm strategies targeting *Candida glabrata* increasingly focus on membrane-active compounds capable of disrupting biofilm formation and mature biofilm structure. Building on previous work identifying alkylamidobetaine C9 (AAB C9)—[(3-decanoylmethylamino)propyl]dimethylammonium acetate—and β-escin as antibiofilm agents against *C. glabrata*, this study examined the biochemical and physiological basis of their efficacy in a reference strain (ATCC 90030) and a clinical isolate (2586). Oxidative stress was assessed microscopically after two hours of exposure, and proteome changes were quantified after six hours, for each compound alone and in combination. Fluorescence microscopy showed that AAB C9 and β-escin interacted differently depending on the ROS compartment and strain: in the strain ATCC 90030, the combination antagonized β-escin-induced mitochondrial superoxide, whereas in the multidrug-resistant isolate 2586 it exceeded either agent alone for both cytosolic and mitochondrial superoxide, indicating a strain-dependent, additive interaction. Quantitative proteomics showed that strain genetic background, rather than treatment, was the dominant source of proteome variance. Preranked gene set enrichment analysis revealed that AAB C9 significantly suppressed accumulation of biofilm-formation-associated proteins specifically in isolate 2586, corroborating its phenotypic antibiofilm activity, while β-escin and the combination produced opposing transmembrane-transport signatures, pointing to compound-specific mechanisms underlying their differential antifungal and antibiofilm effects.

## 1. Introduction

Invasive candidiasis remains a major global cause of morbidity and mortality among critically ill and immunocompromised patients, with reported mortality rates of 30–60% despite antifungal therapy [1,2,3]. Although earlier clinical attention was focused predominantly on *Candida albicans*, the epidemiology of invasive candidiasis has progressively shifted toward non-*albicans Candida* species, which are characterized by greater antifungal resistance, increased persistence in healthcare environments, and higher diagnostic complexity [2,3].

*Candida glabrata*, recently renamed *Nakaseomyces glabratus*, is classified as a high-priority fungal pathogen [4] and has emerged as a leading cause of bloodstream, oral, vaginal, urinary tract, and deep-seated infections, including intra-abdominal abscesses, in immunocompromised individuals. Historically regarded as a low-virulence opportunistic yeast, *C. glabrata* is now recognized as a predominant agent of candidemia and invasive candidiasis, particularly among elderly, critically ill, and previously azole-exposed patients. While reduced azole susceptibility was long the principal therapeutic concern, recent evidence demonstrates rising dual azole–echinocandin resistance, adaptive microevolution during antifungal therapy, and biofilm-associated tolerance mechanisms that contribute to recurrent infections [5,6,7,8]. Resistance to azoles is commonly mediated by transcriptional regulatory changes and efflux pump activity, whereas echinocandin resistance is most frequently linked to hotspot mutations in *FKS* genes and adaptive cell-wall remodeling [6,7,9].

Proteomics has become a valuable tool for dissecting how *C. glabrata* establishes infection, forms biofilm or resists antifungal therapy. A series of seminal publications identified 37 membrane proteins regulated by exposure to clotrimazole and implicated the transcription factor PDR1 (controlling ~20 of them) and the drug: H^+^ antiporters TPO1_1/TPO1_2 in azole resistance [10]. This was supported by an analogous study on 5-flucytosine uncovered 32 regulated membrane proteins, with PDR1 controlling ~50% of the response and the transporters FLR1/FLR2 mediating 5- flucytosine resistance [11]. The first global characterization of the *C. glabrata* biofilm matrix proteome identified pH-dependent protein sets (51 acidic-specific, 206 neutral-specific, 236 shared), implicating mannan/β-glucan metabolism, epithelial adhesins, yapsins, and moonlighting proteins, with PDR1 as a putative biofilm-proteome regulator [12]. Recently, cell surface proteins of *C. glabrata* blastospores and biofilm were studied by the trypsin shaving approach combined with LC–MS/MS identification. The comparison provided data on pronounced strain- and condition-dependent differences in surface protein composition, encompassing adhesins, yapsin proteases and selected moonlighting proteins, such as enzymes of the glycolysis [13]. Given the considerable strain-to-strain variability in virulence traits, including azole resistance and biofilm-forming capacity (e.g., hyperadherence), proteomic studies will continue to provide important insights into the biology of *C. glabrata* and facilitate the identification of new therapeutic targets.

Contemporary approaches in molecular medicine now enable targeted bioengineering at the cellular level. By introducing carefully selected active compounds, it is possible to induce proteome alterations that simultaneously reduce adhesion and enhance eradication of multidrug-resistant fungal strains. Such investigations typically involve the addition of an appropriate chemical entity or mixture—most often surfactants such as combinations of β-escin and alkylamidobetaines (AABs) [14], followed by detailed proteomic analysis focused on proteins involved in biofilm formation.

Alkylamidobetaines constitute a promising class of mild antimicrobial surfactants, particularly when employed in synergy with other agents possessing complementary activity. These compounds have already been incorporated into modern formulations, including hair-care products enriched with natural ingredients such as plant extracts. Exploration of AAB synergy with β-escin aligns with the current trend toward multifunctional, ‘tailor-made’ products designed with specific, dedicated functionalities. Chemically, AABs are surfactants featuring a tertiary amide linker, a stable quaternary nitrogen atom, and a hydrolyzable or biodegradable moiety [15]. β-Escin is a natural triterpene saponin surfactant isolated from the seeds of the horse chestnut tree. Earlier studies on newly synthesized AABs, i.e., [(3-alkanoylmethylamino)propyl]dimethylammonium acetates (C_n_TMDAB, *n* = 9, 11, 13 and 15), demonstrated that the C9-substituted derivative exhibited the highest antibiofilm efficacy against *C. glabrata*; however, the effects of combining these compounds remain poorly characterized [14].

Despite their potential [14], the effects of AABs on fungal pathogens, especially *C. glabrata*, remain insufficiently explored. In particular, there is a lack of comprehensive studies addressing their impact at the molecular level, including changes in the cellular proteome and stress response pathways. Understanding how these compounds influence protein expression, oxidative stress, and cellular homeostasis is essential for elucidating their mechanisms of action. Furthermore, data on their ability not only to prevent biofilm formation but also to disrupt established biofilms are still limited.

Therefore, the present study aims to evaluate the effects of [(3-decanoylmethylamino)propyl]dimethylammonium acetate (AAB C9) and β-escin on both reference and clinical strains of *C. glabrata*. The investigation focuses on multiple aspects of fungal physiology and pathogenicity, including alterations in the proteome, and evaluation of induced stress. By integrating proteomics and oxidative stress analysis, this study seeks to provide a comprehensive understanding of the biological activity of AABs against multidrug-resistant, biofilming *C. glabrata*.

The findings of this work may contribute to the development of innovative strategies for controlling fungal infections, particularly those associated with biofilms and medical devices. Moreover, they may support the design of novel biomaterials and therapeutic approaches that incorporate AABs as effective antiadhesive and antibiofilm components, ultimately improving the management of difficult-to-treat fungal infections.

## 2. Results

### 2.1. Fluorescence Microscopy

**Figure 1:**
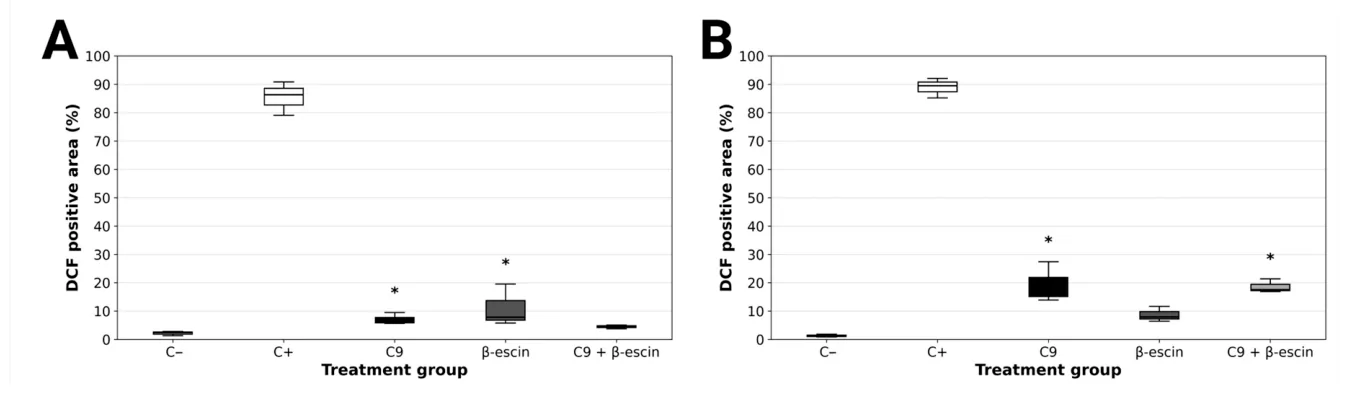
Fluorescence area of DCF (2′,7′-dichlorofluorescein) showing intracellular ROS presence in *C. glabrata* strains ATCC 90030 (**A**) and 2586 (**B**) after treatment with AAB C9, β-escin, and their combination. Control treatments were with PBS (C−) or 25% ethanol (C+). Increased fluorescence intensity indicates elevated oxidative stress. Boxes represent median (*n* = 3) and IQR. Asterisks mark values which differ significantly from the control according to Kruskal–Wallis test followed by Dunn’s post hoc test (*p* < 0.05).

**Figure 2:**
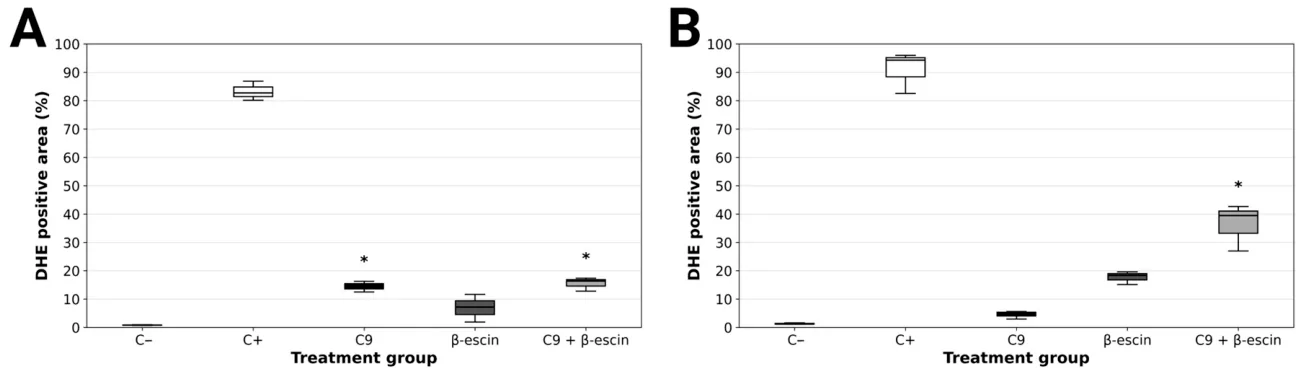
Fluorescence area of DHE showing intracellular presence of superoxide anion (O_2_•^−^) in *C. glabrata* strains ATCC 90030 (**A**) and 2586 (**B**) after treatment with AAB C9, β-escin, and their combination. Control treatments were with PBS (C−) or 25% ethanol (C+). Increased fluorescence intensity indicates elevated oxidative stress. Boxes represent median (*n* = 3) and IQR. Asterisks mark values which differ significantly from the control according to Kruskal–Wallis test followed by Dunn’s post hoc test (*p* < 0.05).

**Figure 3:**
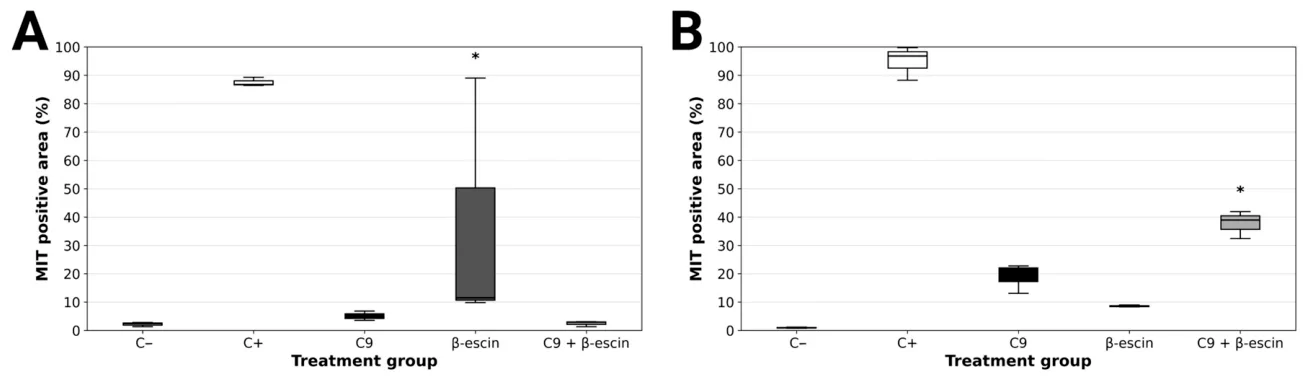
Fluorescence area of MitoSOX™ Red showing mitochondrial presence ROS in *C. glabrata* strains ATCC 90030 (**A**) and 2586 (**B**) after treatment with AAB C9, β-escin, and their combination. Control treatments were with PBS (C−) or 25% ethanol (C+). Increased fluorescence intensity indicates elevated level of ROS in mitochondria. Boxes represent median (*n* = 3) and IQR. Asterisks mark values which differ significantly from the control according to Kruskal–Wallis test followed by Dunn’s post hoc test (*p* < 0.05).

**Figure 4:**
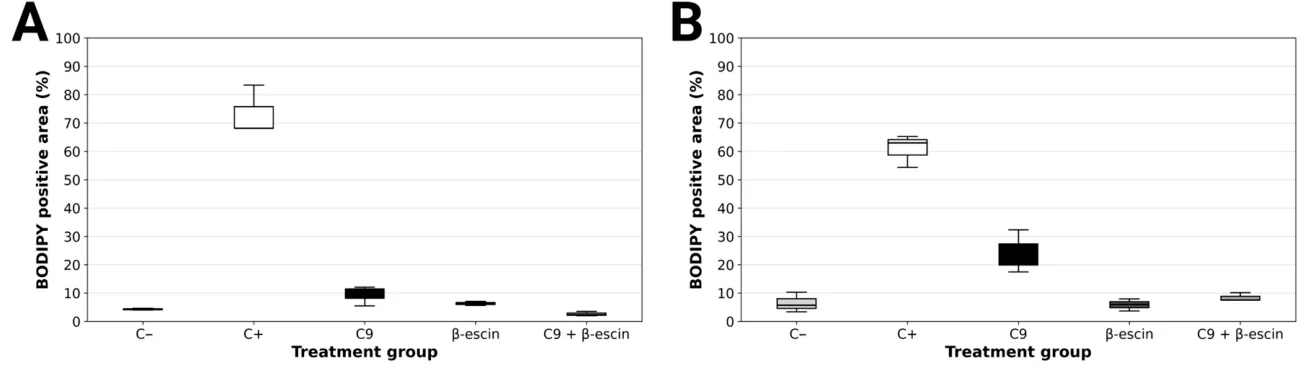
Fluorescence area of BODIPY fluorescent sensor showing presence of lipid peroxidation in *C. glabrata* strains ATCC 90030 (**A**) and 2586 (**B**) after treatment with AAB C9, β-escin, and their combination. Control treatments were with PBS (C−) or 25% ethanol (C+). Increased fluorescence intensity reflects oxidative damage to membrane lipids following the treatment. Boxes represent median (*n* = 3) and IQR. Treated groups were assessed for significant differences from their respective control group using the Kruskal–Wallis test followed by Dunn's post hoc test (*p* < 0.05); none of the groups showed a significant difference.

Fluorescence microscopy assays were performed to evaluate the capacity of AAB C9 (1 µM), β-escin (½ MIC_90_), and their combination to induce oxidative stress in planktonic cells of the reference strain *C. glabrata* ATCC 90030 and the multidrug-resistant clinical isolate 2586. Four complementary fluorescent probes were used: 2′,7′-Dichlorodihydrofluorescein (DCF) for general intracellular reactive oxygen species (Figure 1), Dihydroethidium (DHE) for superoxide anion (Figure 2), MitoSOX™ Mitochondrial Superoxide Indicator (MIT) for mitochondrial superoxide (Figure 3), and BODIPY 581/591 C11 for lipid peroxidation (Figure 4). The microphotographs were evaluated by a computer-assisted image analysis in order to obtain quantitative results. This produced the percentage of alive cells which exhibited specific probe fluorescence in the given treatment. β-Escin alone was the strongest inducer of mitochondrial superoxide, producing the highest MIT-positive reaction in the reference strain ATCC 90030 (Figure 3A). In contrast, AAB C9 alone and the C9 + β-escin combination generated only low mitochondrial ROS levels in this strain, comparable to the negative control (Figure 3A). In the clinical isolate 2586, the combination of C9 and β-escin was the most potent inducer of both superoxide anion (highest DHE-positive reaction; Figure 2B) and mitochondrial ROS (highest MIT-positive reaction; Figure 3B).

The C9 + β-escin combination consistently produced the lowest general ROS level (DCF-positive reaction) in the reference strain ATCC 90030 (Figure 1A). In the clinical isolate 2586, however, AAB C9 alone induced a visibly higher DCF-positive reaction than either β-escin alone or the combination (Figure 1B). All three treatments resulted in minimal lipid peroxidation (BODIPY-positive reaction) in the reference strain, similarly to the negative control (Figure 4A). In the clinical isolate, AAB C9 alone produced a modestly elevated BODIPY signal, whereas β-escin alone and the combination kept lipid peroxidation at low levels (Figure 4B).

The interaction between AAB C9 and β-escin was neither uniformly additive nor uniformly antagonistic but instead depended on both the cellular compartment probed and the strain tested. In the reference strain ATCC 90030, the combination never exceeded the more active single agent at any endpoint: it was significantly lower than both single agents for general ROS (DCF; Figure 1A), matched the weaker of the two single-agent effects for cytosolic superoxide (DHE; Figure 2A), and abolished the strong mitochondrial superoxide induction seen with β-escin alone, returning MIT-positive signal to near-baseline levels (Figure 3A).

In the clinical isolate 2586, the same combination behaved in the opposite direction for the two superoxide-sensitive probes: DHE- and MIT-positive signal under the combination exceeded the arithmetic sum of the two single-agent effects (Figure 2B and Figure 3B), consistent with a greater-than-additive, potentiating interaction. However, for general ROS and lipid peroxidation in this isolate, the combination did not exceed AAB C9 alone and in the case of lipid peroxidation was suppressed to the level of β-escin alone despite C9 alone producing the highest BODIPY signal of the three treatments (Figure 4B). This suggests that β-escin can antagonize a C9-specific effect even in the strain where it potentiates C9 at the mitochondrial level.

### 2.2. Proteomics

The reference proteome UP000002428 from UniProt contains 5198 unique genes found in the proteome set. Our proteome analysis of planktonic *C. glabrata* treated with AAB C9 and β-escin resulted in the annotation of 4461 quantified proteins across the eight experimental groups. This represents approximately 85.8% of the reference proteome. This number is similar to the one of the earliest panproteomic studies by [16], reporting the use of high-resolution Fourier-transform mass spectrometry applied to study *C. glabrata* and validating 4421 of 5283 predicted coding genes (≈83% of the genome).

**Figure 5:**
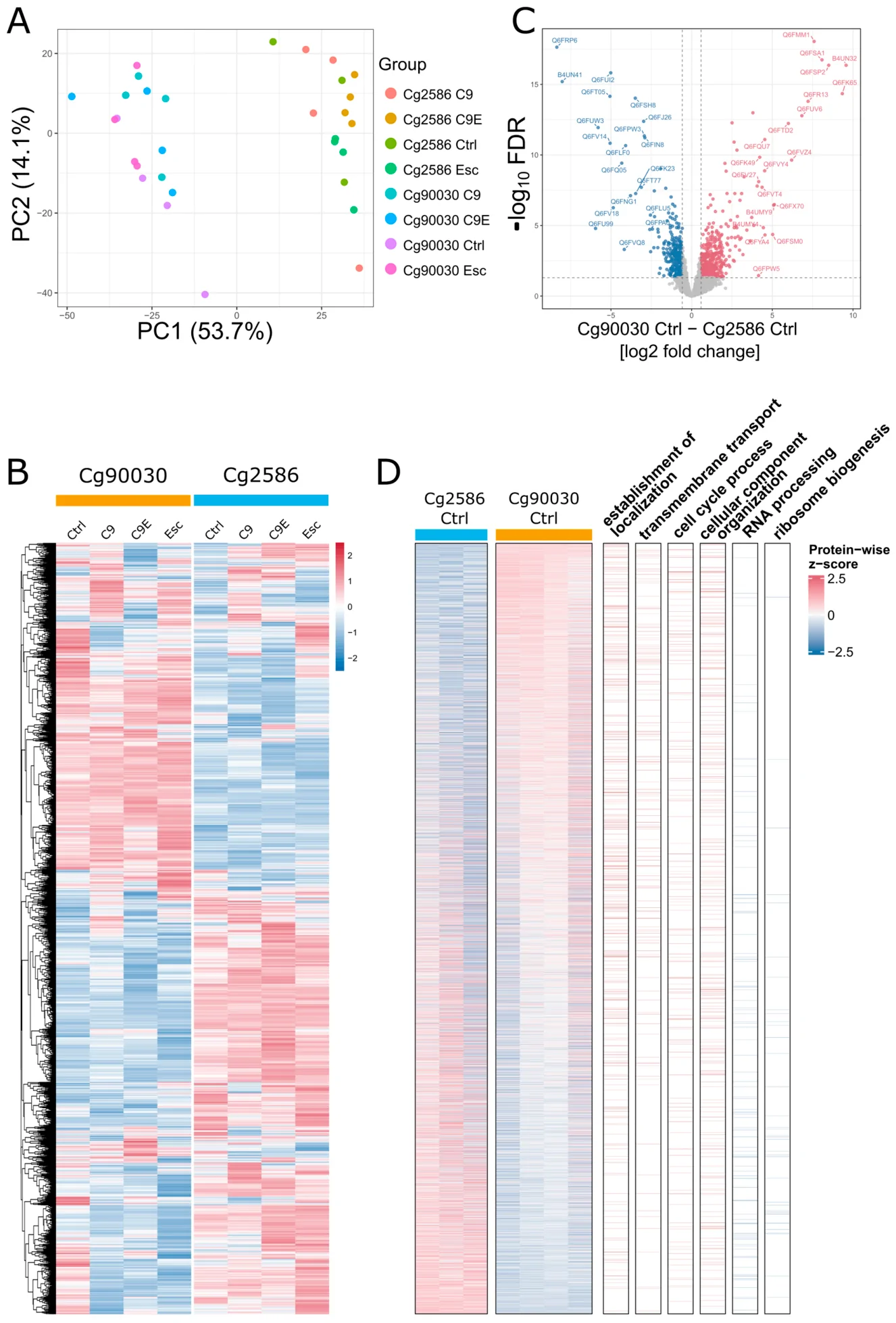
Proteome profiling of *C. glabrata* reference strain ATCC 90030 and the clinical isolate 2586 after treatment with AAB C9 and β-escin. (**A**) Principal component analysis of the normalized and imputed protein-abundance matrix. Each point represents one biological replicate, and colors indicate experimental groups. Percentages denote the variance explained by the first two principal components. (**B**) Heatmap of all quantified proteins. Values represent group-averaged processed log_2_ abundances, scaled separately for each protein. Rows were hierarchically clustered using the Euclidean distance and the complete-linkage method (*n* = 3–4 biological replicates). (**C**) Volcano plot comparing the protein abundances under control conditions of reference strain ATCC 90030 and clinical isolate 2586. Proteins with FDR < 0.05 and |log_2_ fold change| > 0.58 are shown in red when more abundant in reference strain ATCC 90030 and in blue when more abundant in clinical isolate 2586; nonsignificant proteins are gray. Selected strongly regulated proteins are labeled by UniProt accession number. (**D**) Protein-wise z-scores are shown for the individual control replicates of isolate 2586 and strain ATCC 90030. Proteins were ordered by the moderated *t*-statistic for the strain ATCC 90030 versus the isolate 2586 comparison. The adjacent annotation tracks indicate membership in selected nonredundant Gene Ontology Biological Process terms identified by fast gene set enrichment analysis.

The proteomes of the two strains grown in control conditions (mock treatment) were compared by Primary Component Analysis, hierarchical clustering and fast gene set enrichment analysis for selection of Gene Ontology terms enrichment. The results are presented in Figure 5. From the results, it is evident that the primary component explaining most of the variance in the dataset is the genotype/strain of the studied *C. glabrata* (Figure 5A). The PC2 (14.1%) gives a partial within-strain separation of control from treated replicates especially for ATCC 90030, where the Ctrl points sit apart from treatment points (C9/C9E/Esc) (Figure 5A). The row-clustered heatmap (Figure 5B) of group-averaged abundances resolved two large strain-specific blocks: a set of proteins more abundant in ATCC 90030 vs. 2586, and a set more abundant in 2586 vs. ATCC 90030. This illustrates the same structure of the data as panel A, but at the protein level.

The volcano plot in Figure 5C depicts contrast between strain ATCC 90030 Ctrl vs. strain 2586 Ctrl. The points are arranged in the form of a large, symmetric cone.

Under standard growth conditions (mock treatment), we identified 464 proteins with significantly higher abundance in strain ATCC 90030 and 343 proteins with significantly higher abundance in strain 2586 (Figure 5C). The two most significant hits higher in ATCC 90030 are B4UN32 (AWP3b) and Q6FK65 (SIR3), whereas those higher in 2586 are Q6FUI2 (GVI51) and B4UN41 (uncharacterized protein). Gene set enrichment analysis (fgsea) using Gene Ontology (GO) Biological Process terms showed that proteins more abundant in ATCC 90030 were enriched in processes related to establishment of localization, transmembrane transport, cell cycle processes, and cellular component organization. In contrast, proteins more abundant in isolate 2586 were primarily associated with RNA processing and ribosome biogenesis (Figure 5D).

**Figure 6:**
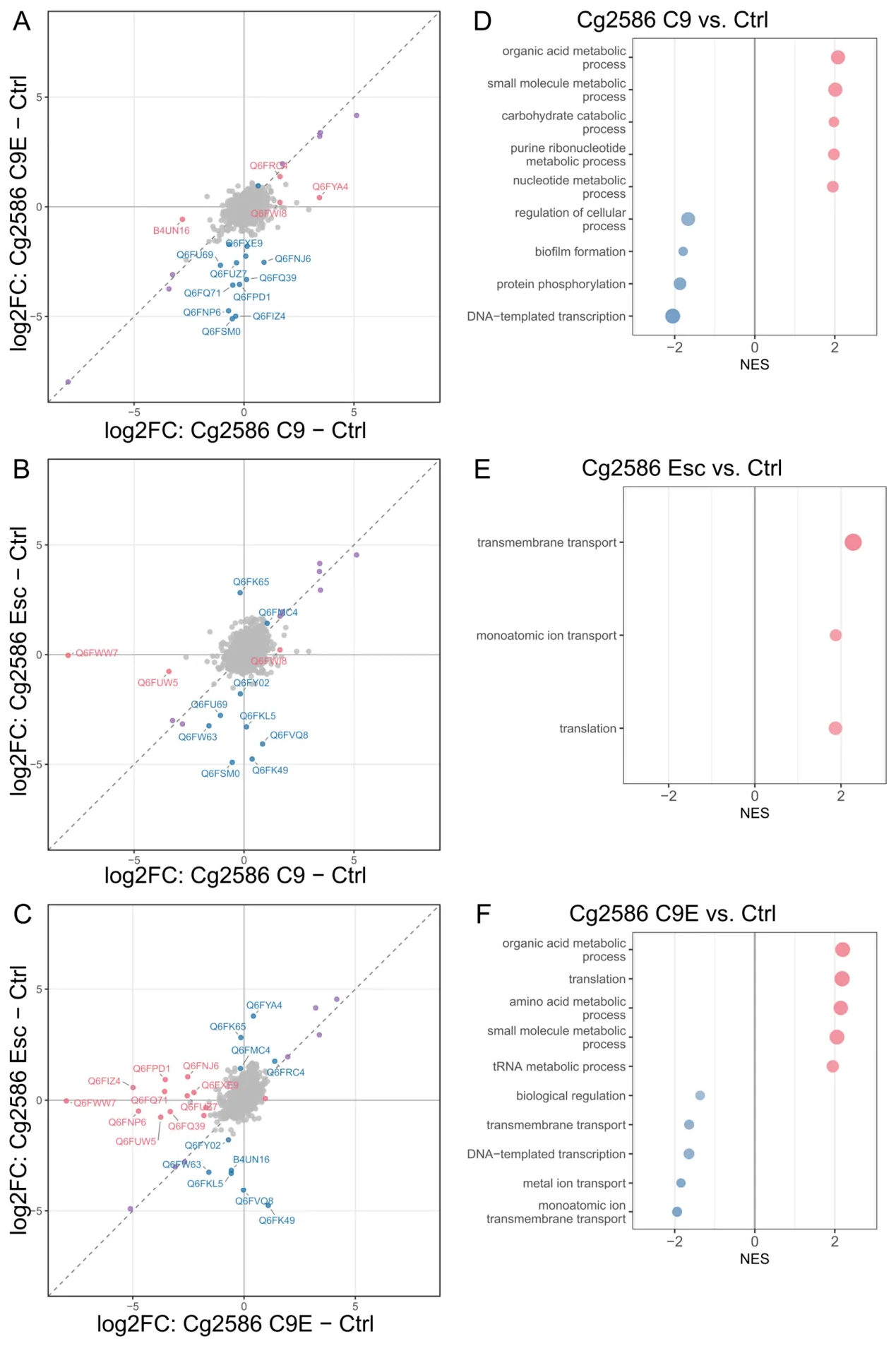
Comparison of treatment-induced proteomic responses in clinical isolate 2586. (**A**–**C**) depict pairwise comparisons of protein-level log_2_ fold changes for the three treatments relative to isolate 2586 control conditions: AAB C9 versus AAB C9 + β-escin (**A**), AAB C9 versus β-escin (**B**), and AAB C9 + β-escin versus β-escin alone (**C**). Each point represents one protein. Proteins significantly different exclusively in the contrast shown on the x-axis are red, those significant exclusively in the y-axis contrast are blue, proteins significant in both contrasts are purple, and proteins significant in neither contrast are gray. Significance was defined as FDR < 0.05 and |log_2_ fold change| > 0.58. Proteins showing a treatment-specific response are labeled by accession number. Dashed lines indicate identical fold changes in both contrasts. (**D**–**F**) visualize preranked fast gene set enrichment analysis results of Gene Ontology Biological Process terms for AAB C9 (**D**), β-escin (**E**), and AAB C9 + β-escin (**F**) relative to isolate 2586 in control conditions. Proteins were ranked by the moderated *t*-statistic from the corresponding limma contrast. Significant terms were reduced to nonredundant representatives, and up to five terms with highest positive nonredundant enrichment score (NES) and up to five with the lowest negative NES were displayed for each contrast. Positive NES values indicate enrichment toward the treatment, whereas negative values indicate enrichment toward the isolate 2586 control conditions. Red and blue denote positive and negative NES, respectively, and larger point means a lower FDR. Common NES and FDR scales were used across all enrichment panels.

Next, we focused on the analysis of the treatment-specific response of clinical strain 2586 (Figure 6A–C). Pairwise comparison of the proteomic responses to AAB C9, β-escin, and their combination in isolate 2586 showed that most treatment-responsive proteins were driven predominantly by β-escin rather than by C9: proteins significantly altered under the C9 + β-escin combination were, with few exceptions, also significantly altered by β-escin alone, whereas C9 alone produced comparatively few significant changes at the protein level (Figure 6A–C).

Gene set enrichment analysis suggests that AAB C9 alone predominantly upregulated proteins involved in organic acid, small-molecule, and carbohydrate/nucleotide metabolic processes, while significantly suppressing proteins involved in biofilm formation, protein phosphorylation, and DNA-templated transcription (Figure 6D). The treatment with β-escin alone upregulated proteins responsible for transmembrane transport, ion transport, and translation (Figure 6E). The combined treatment retained the AAB C9-like upregulation of proteins related to metabolic processes but added emphasis toward translation and tRNA processing (Figure 6F). Notable downregulation of proteins involved in biofilm formation was specific to the AAB C9 treatment. The nine out of twenty proteins in *C. glabrata* 2856 with the GO term are summarized in Table 1.

**Figure 7:**
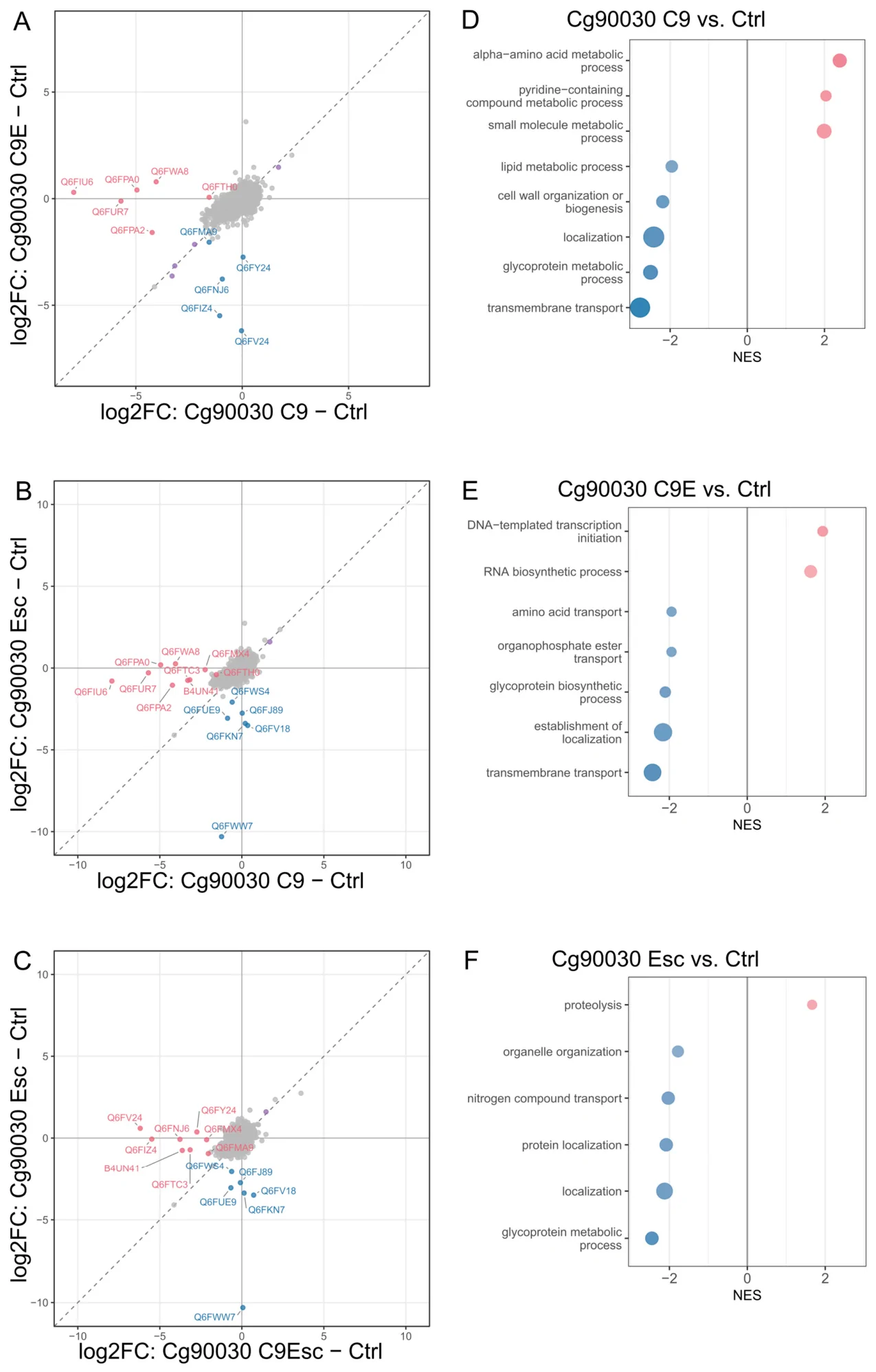
Comparison of treatment-induced proteomic responses in reference strain 90030. (**A**–**C**) depict pairwise comparisons of protein-level log_2_ fold changes for the three treatments relative to reference strain 90030 control conditions: AAB C9 versus AAB C9 + β-escin (**A**), AAB C9 versus β-escin (**B**), and AAB C9 + β-escin versus Esc (**C**). Each point represents one protein. Proteins significant exclusively in the contrast shown on the x-axis are red, those significant exclusively in the y-axis contrast are blue, proteins significant in both contrasts are purple, and proteins significant in neither contrast are gray. Significance was defined as FDR < 0.05 and |log_2_ fold change| > 0.58. Proteins showing a treatment-specific response are labeled by accession number. Dashed lines indicate identical fold changes in both contrasts. (**D**–**F**) show visualize preranked fast gene set enrichment analysis results of Gene Ontology Biological Process terms for AAB C9 (**D**), AAB C9 + β-escin (**E**), and β-escin (**F**) relative to reference strain 90030 control conditions. Proteins were ranked by the moderated *t*-statistic from the corresponding limma contrast. Significant terms were reduced to nonredundant representatives, and up to five terms with positive NES and five with negative NES were displayed for each contrast. Positive NES values indicate enrichment toward the treatment, whereas negative values indicate enrichment toward strain 90030 control conditions. Red and blue denote positive and negative NES, respectively, and a larger point means a lower FDR. Common NES and FDR scales were used across all enrichment panels.

Pairwise comparison of the proteomic responses to AAB C9, β-escin, and their combination in reference strain ATCC 90030 showed treatment-specific responses (Figure 7A–C). Most proteins significantly altered by AAB C9 + β-escin were not significantly altered by C9 alone (Figure 7A), and the response to β-escin likewise showed limited overlap with that induced by C9 alone (Figure 7B).

Gene set enrichment analysis showed that AAB C9 predominantly upregulated proteins involved in α-amino acid, pyridine-containing compound, and small-molecule metabolic processes, and downregulated proteins important for lipid metabolism, cell wall organization or biogenesis, localization, glycoprotein metabolism, and transmembrane transport (Figure 7D). The treatment with β-escin alone increased the abundance of proteins related to proteolysis, and decreased the abundance of proteins important for organelle organization, nitrogen compound transport, protein localization, localization, and glycoprotein metabolism (Figure 7F). The combined AAB C9 + β-escin treatment showed a different functional profile, by increasing the abundance of proteins incolved in DNA-templated transcription initiation and RNA biosynthetic processes, and decreasing the abundance of proteins associated with amino acid transport, organophosphate ester transport, glycoprotein biosynthesis, establishment of localization, and transmembrane transport (Figure 7E). Thus, in ATCC 90030 AAB C9 predominantly affected the abundance of proteins involved in metabolic processes, while β-escin was associated with proteolysis and intracellular organization, whereas the combined treatment produced a distinct response characterized by altered abundance of proteins associated with transcriptional and RNA-related processes together with altered transport and localization.

Supplementary Tables S1–S4 are within an .xlsx file with and contain processed data from each step of the proteomic analysis: DIA-NN protein-group abundance matrix, protein-abundance matrix used for statistical analysis, results of limma-based differential protein-abundance analysis, and the results of FGSEA analysis.

## 3. Discussion

The interference with biofilm formation and promotion of the disruption of mature biofilms has been demonstrated previously, highlighting the potential of [(3-decanoylmethylamino)propyl]dimethylammonium acetate (AAB C9) and β-escin as antibiofilm agents [14]. This study, by the application of microscopical evaluation of oxidative stress and proteome modifications induced in *C. glabrata*, sought to shed more light on the biochemical and physiological nature of their efficacy as antibiofilm agents.

Both applied compounds are expected to interfere with biological membranes. [(3-alkanoylmethylamino)propyl]dimethylammonium acetates were synthetized recently. Based on their structure, the most probable reason for the previously reported antifungal activity is the interaction with phospholipids of the cell membrane as well as the ability to destabilize membrane proteins and the disruption of protein interactions due to the presence of the suractant’s hydrolysis products, i.e., decanoic (capric) acid or quaternary amine betaine-bearing motif—i.e., (methylaminopropyl)dimethylammonium acetate. As much as 100 μg/mL β-escin leads to full hemolysis of human erythrocytes [14]. As demonstrated by Sreij et al. [17], the incorporation of β-escin molecules into a model bilayer increases the bilayer fluidity and β-escin molecules phase-segregate into saponin-rich and saponin-poor domains in the membrane. With increasing β-escin concentration, vesicles start to become unstable in solution and agglomerate due to the formation of larger domains. At concentrations far above the CMC of β-escin, the model membrane is solubilized [18,19]. β-Escin interacts with cholesterol in membranes and this is probably true for fungal membranes which contain ergosterol. Wojciechowski et al.’s [20] results suggest that the presence of 20% cholesterol dramatically sensitizes high-curvature bilayers to saponins. For *C. glabrata* these might include tubules of the endoplasmic reticulum and the Golgi, the bud neck during cell division, the mitochondrial inner membrane, and various types of vesicles. One could expect these structures to be affected by the treatment leading to destabilization of membranes, ROS leakage or changes in protein–protein interactions, especially membrane-associated proteins. In our experiments, cells remained intact, showing membrane stability in the presence of β-escin and β-escin combined with AAB C9. Collectively, our results indicate that β-escin and AAB C9 interact differently depending on the ROS compartment and the genetic background of the strain, ranging from antagonistic (mitochondrial superoxide in ATCC 90030; lipid peroxidation in isolate 2586) to apparently additive/neutral (cytosolic ROS in ATCC 90030) to potentiating (cytosolic and mitochondrial superoxide in isolate 2586).

The observed changes in membrane properties and mitochondrial ROS production are expected to propagate beyond the membrane itself, affecting cellular pathways that maintain protein and organelle integrity. Oxidative damage together with altered membrane organization can disrupt protein folding, compromise the activity of membrane-associated protein complexes, and increase the demand for chaperone-mediated quality control. In agreement with this model, several proteins involved in proteostasis, membrane-associated proteins and cellular stress responses exhibited altered abundance following AAB C9 and β-escin treatment. The observed low number of proteins which changed their abundance more then 2-fold relative to the control, might be explained by the rather low concentration of the compounds and the pleiotropic nature of the expected interactions with cellular membranes.

The comparison of the control proteomes demonstrated that strain background was the principal source of variation in the dataset, with the separation between ATCC 90030 and clinical isolate 2586 clearly exceeding the variation associated with treatment (Figure 5A,B). This finding indicates that the two *C. glabrata* strains entered the experiments in substantially different physiological states and provides an important context for interpreting their treatment responses. At the same time, the treatment comparisons demonstrated that AAB C9 and β-escin do not induce equivalent proteomic responses in ATCC 90030 and clinical isolate 2586.

The stark strain-dependent differences were also evident at the individual protein level. Among the most strongly differentiated proteins, AWP3b was more abundant in ATCC 90030, whereas GVI51 and the uncharacterized protein (B4UN41) sharing high similarity to hyphally-regulated cell wall protein N-terminal domain-containing protein were more abundant in isolate 2586 (Figure 5C). The higher abundance of AWP3b in the reference strain is of particular interest given the role of cell-wall adhesins in *C. glabrata* adhesion and biofilm formation. Hyphally-regulated cell wall protein N-terminal domain-containing proteins are known to play an important role in the formation of biofilm and virulence of *C. glabrata* [21,22]. The changes in abundance of an individual membrane protein associated with adhesion and biofilm formation cannot by itself be taken as evidence for differences in adhesive capacity between the two studied strains of *C. glabrata*.

A particularly notable finding in isolate 2586 was the negative enrichment of biofilm formation following treatment with AAB C9, which was not observed for β-escin or the combined treatment (Figure 6D). The proteins contributing to this annotation included several transcriptional and chromatin-associated regulators, including EFG1, SNF2, HAA1, SIR4, ZAP1, CST6, MSS11, UPC2A and RIF1. The seminal paper by Riera et al. [23] on the genetic mutant screening of disturbed biofilm formation in *C. glabrata* described and discussed three of the nine proteins negatively enriched in the treatment, which are listed in Table 1. They have shown that *EPA6* expression is positively regulated by the chromatin remodeling Swi/Snf complex, whereas transcription factor Cst6p is a negative regulator of *EPA6* transcription in a Sir4p-dependent manner [23]. Another interesting finding among the negatively enriched was EFG1, which has been identified through a genetic screen by Cavalheiro et al. [24]. The transcription factor, alongside TEC1, is required for biofilm formation and adhesion to vaginal epithelium. In *C. glabrata*, transcription factors EFG1 and TEC1, but not their paralogues EFG2 and TEC2, play a role in biofilm formation. The role of EFG2 remains unknown. These results are in line with the antibiofilm activity previously attributed to AAB C9 and through putative downregulation of these proteins provides a plausible bridge between our proteomics and the phenotypic observation of biofilm inhibition/eradication [14].

## 4. Materials and Methods

### 4.1. Reagents

The liquid chromatography reagents (acetonitrile, water, methanol) were of the highest mass spectrometry grade and were supplied by Biosolve BV (Valkenswaard, The Netherlands). Trifluoroacetic acid was obtained from Merck (Darmstadt, Germany). Reagents for microscopic preparate staining, in particular the molecular probes, were purchased from Merck (Darmstadt, Germany) orThermo Fisher Scientific (Waltham, MA, USA) as declared below. All other chemicals and solvents, used for [(3-decanoylmethylamino)propyl]dimethylammonium acetate (AAB C9) synthesis, were obtained from Sigma-Aldrich, Alfa Chemistry or Avantor Performance Materials.

### 4.2. The Preparation and Chemical Characterization of [(3-Decanoylmethylamino)propyl]dimethylammonium Acetate (AAB C9)

[(3-decanoylmethylamino)propyl]dimethylammonium acetate (AAB C9, abbreviated also as C_9_TMDAB) was synthesized by the quaternization (with sodium chloroacetate) of amideamine derivative—N-[3-(dimethylamine)propyl]-N-methyldecylamide—obtained starting from N,N,N′-trimethyl-1,3-propanediamine and decanoyl chloride. The whole procedure—the general synthetic route for C_n_TMDAB compounds—is described in detail in our previous paper [14] as well as in Polish patent PL 237430 B1. The obtained compound was purified by means of repeated crystallization from a methanol-ethyl acetate mixture at −20°C. Chemical structure and purity of the synthesized [(3-decanoylmethylamino)propyl]dimethylammonium acetate was confirmed by elemental analyses (Vario EL cube, Elementar Analysensysteme GmbH, Langenselbold, Germany), ESI-MS (micrOTOF-Q instrument; Bruker Daltonics, Bremen, Germany) and ^1^H NMR (Bruker AMX-500 spectrometer, solution in CDCl_3_), indicating high quality of the desired product—see [14] for details. The obtained surfactant was characterized by excellent aqueous solubility—see Krafft points measurements in [14].

### 4.3. Strains and Growth Conditions

The *Candida glabrata* reference strain (ATCC 90030) was purchased from the American Type Culture Collection (LGC France SARL, Strasbourg, France), and 1 multidrug-resistant clinical strain (2586) was obtained from the collection of the Department of Pharmaceutical Microbiology and Parasitology, Faculty of Pharmacy, Wroclaw Medical University. The strain 2586 was isolated from a patient of the Clinical University Hospital in Wroclaw from the vagina and its resistance profile was determined in a previous study [14].

Yeasts were cultured for 24 h in yeast extract–peptone–glucose (YPG) medium (1% yeast extract, 1% peptone, 2% glucose; pH 5.6) at 37 °C. Following incubation, cultures were centrifuged and resuspended in fresh YPG medium or phosphate-buffered saline (PBS, pH 7.2) to obtain the desired optical density. All culture media used were produced by BD Difco (Mississauga, ON, Canada) in powder form. They were prepared according to the manufacturer’s instructions.

### 4.4. Preparation of Cell Suspensions

For the pre-culture, cells were inoculated into liquid medium and incubated at 37 °C for 24 h under aerobic conditions. Following incubation, cells were harvested by centrifugation and washed twice with sterile PBS. The cell pellet was subsequently resuspended in PBS. Cell density was adjusted to 2.0 McFarland standard using a densitometer, corresponding to approximately (4 × 10^6^ CFU/mL). Standardized suspensions were incubated at 37 °C for 48 h without the addition of tested compounds to allow stabilization and adaptation.

### 4.5. Treatment Conditions

All experiments were conducted under standardized and controlled conditions. Independent biological replicates and appropriate controls were included to ensure reproducibility and reliability of the results. Cells incubated in PBS under identical experimental conditions served as negative controls.

#### 4.5.1. AAB C9 Treatment

After the initial 48 h incubation, AAB C9 was added at the final concentration of 1 μM. Samples were then incubated for an additional 6 h at 37 °C. Following exposure, cells were harvested by centrifugation and washed twice with PBS to remove residual compounds.

#### 4.5.2. β-Escin Treatment

β-Escin was applied at concentrations corresponding to ½ MIC_90_ based on previously determined values [14]. The MIC_90_ values were 128 µg/mL for ATCC 90030 and 256 µg/mL for strain 2586. Incubation was carried out for 6 h at 37 °C, followed by washing with PBS.

#### 4.5.3. Combined Treatment

For combination studies, AAB C9 and β-escin were added simultaneously at the concentrations described above. Samples were incubated for 6 h at 37 °C and subsequently washed twice with PBS prior to the harvest by centrifugation.

### 4.6. Fluorescence Microscopy

Imaging was conducted with an Olympus BX51 fluorescence microscope (Tokyo, Japan) fitted with a 10× Plan APO objective and an Orca Flash 40 camera (Hamamatsu, Hamamatsu City, Japan), using a scale bar of 100 μm.

#### 4.6.1. Cell Imaging

Yeast cells (OD = 0.6 in PBS) and 4 μM of Calcofluor White M2R (Merck, Darmstadt, Germany) were incubated for 120 min. Imaging was performed with the use of filter 43 HE (λ_ex_ = 510 nm, λ_em_ = 580 nm).

#### 4.6.2. Oxidative Stress Analysis

Blastospores were obtained from 24 h cultures grown at 37 °C in YPG medium, harvested by centrifugation, washed twice with PBS, and resuspended in PBS to an optical density of OD_600_ = 0.6. Cell suspensions were exposed to the following conditions: AAB C9 at a final concentration of 1 µM; β-escin at concentrations corresponding to ½ × MIC_90_ (i.e., 64 µg/mL for ATCC 90030 and 128 µg/mL for strain 2586) [14]; a combination of AAB C9 and β-escin at the concentrations specified above. Control samples without treatment (label C−) were included in all experiments. Positive control samples (label C+) used an established stressor, ethanol, in the final concentration of 25% applied for four hours. Where indicated, cells were incubated with the tested compounds for 4 h at 37 °C.

##### Detection of General Intracellular Oxidative Stress

General intracellular oxidative stress was assessed using 2,7-dichlorofluorescin diacetate (DCFH-DA; Merck, Darmstadt, Germany). Cell suspensions (OD_600_ = 0.6 in PBS) were incubated with 2 µM DCFH-DA for 120 min at 37 °C in the dark. After incubation, cells were washed twice with PBS to remove excess dye and subsequently exposed to the tested compounds under the conditions described above. The oxidation of non-fluorescent DCFH to fluorescent dichlorofluorescein (DCF) was monitored using a fluorescence microscope (Carl Zeiss Axio Imager M1) equipped with filter set 38 HE (λ_ex_ = 495 nm, λ_em_ = 517 nm). The proportion of fluorescent cells was determined from at least three independent experiments.

##### Detection of Superoxide Anion

The formation of superoxide anion (O_2_•^−^) was evaluated using dihydroethidium (DHE; Merck, Darmstadt, Germany). Cells (OD_600_ = 0.6 in PBS) were incubated with 2 µM DHE for 120 min at 37 °C in the dark, washed twice with PBS, and then treated with the tested compounds. Oxidation of DHE to ethidium resulted in red fluorescence, which was detected using a fluorescence microscope with filter set 43 HE (λ_ex_ ≈ 520 nm, λ_em_ ≈ 610 nm).

##### Detection of Mitochondrial Oxidative Stress

Mitochondrial superoxide production was measured using MitoSOX™ Red mitochondrial superoxide indicator (Thermo Fisher Scientific, Waltham, MA, USA). Yeast cells (OD_600_ = 0.6 in PBS) were incubated with 2 µM MitoSOX Red for 120 min at 37 °C in the dark. After washing twice with PBS, cells were exposed to the tested compounds. Fluorescence was observed using a fluorescence microscope with appropriate filter settings (λ_ex_ ≈ 396 nm, λ_em_ ≈ 580 nm). The fraction of cells exhibiting mitochondrial ROS was quantified.

##### Detection of Lipid Peroxidation

Lipid peroxidation was assessed using BODIPY™ 581/591 C11 (Thermo Fisher Scientific, Waltham, MA, USA). Cells suspended in PBS (OD_600_ = 0.6) were incubated with 2 µM dye for 120 min at 37 °C in the dark, followed by washing twice with PBS. Fluorescence was measured in both reduced (red; λ_ex_ ≈ 581 nm, λ_em_ ≈ 591 nm) and oxidized (green; λ_ex_ ≈ 488 nm, λ_em_ ≈ 510 nm) states.

#### 4.6.3. Computer Analysis of Fluorescence Microscopy Images

Comprehensive qualitative and quantitative computer analyses were performed on the images to estimate the coverage area. Oxidative stress was assessed by calculating the fluorescence intensity of the applied Calcofluor White (MR2)/oxidative stress probes. The resulting images were processed using Fiji/ImageJ software ver. 1.53c (NIH, Bethesda, MD, USA). The process began by generating maximum intensity projections (MIPs) from image stacks, after which the areas of the binary images were transferred into the original MIP images for the Calcofluor White (MR2) and oxidative stress probes channels. Finally, the mean fluorescence intensities for every detected object within the field of view were determined using the Analyze Threshold function in ImageJ version 1.53 (Software: ImageJ from Softonic).

### 4.7. Proteomic Analysis

#### 4.7.1. Sample Preparation

Cells harvested for the proteomic analysis (*n* = 4) were treated with methyl-tert-butylether/methanol mixture to extract polar and lipophilic compounds. For details of the procedure see Chudzik et al. [25]. The residual protein-containing pellet was dissolved in 50 mM TEAB pH 8.5 and urea. Four protein samples per condition were aliquoted to contain 10 µg protein each. Protein concentration in the extracts were determined with Bradford assay following the manufacturer’s protocol (Bio-Rad Protein Assay Dye Reagent Concentrate, Bio-Rad, Hercules, CA, USA). Next, samples were prepared for digestion using the Single Tube Solid Phase Sample Preparation (SP3) method on a KingFisher Apex platform as described before by Voran et al. [26]. After digestion, peptides were desalted using Affinisep Stage Tips 200 µL C18 T2. After initial sequential washing with (i) 50% MeOH, (ii) 80% ACN, 0.1% TFA, and (iii) 0.1% TFA, acidified peptides were loaded on the stage tips. After washing with 0.1% TFA, peptides were eluted twice using 80% ACN, 0.1% TFA.

#### 4.7.2. LC-MS/MS Analysis and Data Processing

Nanoflow LC-MS2 analysis was performed using the Vanquish Neo coupled to an Orbitrap Eclipse (Thermo Fisher Scientific) as described before by Würtz et al. [27]. Peptide separation was achieved using a 120 min linear gradient, starting at 4% solvent B and gradually increasing to 32% over the first 100 min, followed by a further increase to 49% over the next 20 min, and finally a wash step with 99% solvent B. The Orbitrap Tribrid Eclipse was operated in data-independent acquisition (DIA) mode. Full scan precursor spectra were acquired in the centroid mode with 60k resolution, AGC target = 1.2e6, MaxIT = Auto, RF Lens = 30%, mass range = 400–920. Fragment spectra were then recorded using centroid mode at 30,000 resolution, fragmenting 40 variable windows covering the entire precursor mass range, designed to balance precursor density and minimize spectral complexity. Isolated precursors were fragmented in the HCD cell using 27% normalized collision energy, a normalized AGC target of 1 × 10^6^, and a maxIT of 54 ms. The acquired spectra were searched against the UniProt *C. glabrata* (UP000002428, downloaded on Jan 2026), and a customized contaminant database using DIA-NN 2.2.0 [28]. A maximum of 1 missed cleavage and 2 variable modifications were allowed. The fragment ion mass tolerance was set to 10 ppm, and the parent ion mass tolerance to 20 ppm. The match between runs option was enabled, together with protein inference, and the following settings were used: Scoring = ‘Generic’, Proteotypicity = ‘Protein names’, Machine learning = ‘NNs cross-validated’, Cross-run normalization = ‘Off’, Library Generation = ‘IDs, RT and IM Profiling’, and Speed and RAM usage = ‘Optimal results’. Precursor FDR was set to 1%. Trypsin was specified as an enzyme. The following variable modifications were allowed: Oxidation (M), Met-loss (M), whereas Carbamidomethylation (C) was set as a fixed modification. Quantification was done using DIA-NN’s built-in algorithm QuantUMS (high precision) [29].

Due to the low quality of results, one of four biological replicates of the control for strain 2586 was removed from further processing. The remaining three samples were processed as follows.

The mass spectrometry proteomics data have been deposited to the ProteomeXchange Consortium via the PRIDE [30] partner repository with the dataset identifier PXD083719.

#### 4.7.3. Proteomics Data Analysis

Protein-group quantities generated with DIA-NN version 2.2.0 [28] were used as the quantitative input for downstream analysis in R [31]. The dataset comprised 32 samples. Due to a significantly lower number of identified proteins and consequently nearly 40% missing values, one of four biological replicates of the control for strain 2586 was removed from further processing. The remaining 31 samples were processed as follows. Seven groups contained four biological replicates, whereas strain 2586 Ctrl contained three replicates. Subsequent steps did not assume equal group sizes. Proteins groups annotated as common Repository of Adventitious Proteins (cRAP) contaminants were removed. Protein groups were required to be supported by at least one proteotypic peptide sequence and at least two peptide sequences in total (N.Proteotypic.Sequences ≥ 1 and N.Sequences ≥ 2). Empty quantitative entries were treated as missing values. Initial data quality was assessed at the sample level by calculating the number and percentage of quantified protein groups, inspecting log2-intensity distributions, and evaluating the distribution of missing values. These checks were performed before the protein-level reproducibility filter so that sample-specific differences in data completeness could be identified. For statistical analysis, protein-group quantities were log2-transformed. A protein group was retained if it had at least three finite measurements in at least one experimental group. This criterion was evaluated separately for every group and then combined with an ‘at least one group’ rule. Normalization was performed before missing-value imputation. The filtered log2 matrix was first returned to the linear abundance scale, and quantile normalization was applied across all sample columns with the function from preprocessCore [32]. Protein and sample identifiers were restored after normalization, and the normalized quantities were transformed back to log2 scale. Missing values were imputed using a two-stage strategy. In the first stage, putative missing-not-at-random values were defined conservatively as group-complete missing blocks: for a given protein group, every replicate in an experimental group had to be missing. Candidate values were generated by applying imputeLCMD::impute.MinProb to the complete normalized log2 matrix with q = 0.001, tune.sigma = 1.0, and a random seed of 1 [33]. In the second stage, all remaining missing values were imputed with the non-parametric missForest algorithm, which uses iterative random-forest prediction and can accommodate nonlinear relationships among samples [34]. The matrix after group-complete MinProb imputation was supplied to missForest with the package defaults. Principal component analysis was performed on the final log2-transformed, normalized, and imputed protein-abundance matrix. Differential protein abundance was assessed using the limma R package (version 3.68.5) [35] applied to the final log_2_-transformed, normalized, and imputed abundance matrix. All pairwise comparisons between experimental groups were tested. *p*-Values were adjusted using the Benjamini–Hochberg procedure [36], and proteins with an adjusted *p*-value < 0.05 and an absolute log_2_ fold change > 0.58 were considered differentially abundant.

For the heatmaps, we calculated the arithmetic mean of the processed log2-transformed abundance values for each protein within each experimental group. Consequently, the displayed group-level values remained on the same log2 scale used for the limma analysis and correspond to the log2 of the geometric mean abundance on the original scale. Each protein row was centered and scaled separately across group means to produce a row z-score. Constant rows were retained and displayed at zero. Values outside −2.5 to 2.5 were clipped only for color mapping. The heatmap included all quantified proteins, used Euclidean distance and complete-linkage clustering for rows only, and preserved the sample-list condition order without column clustering.

Gene set enrichment analysis was performed with fgsea R package (version 1.38.0) [37,38] using *Candida glabrata* Gene Ontology Biological Process annotations from the *Candida* Genome Database (cgd.gaf.gz) [39,40]. To visualize basal differences between strains 90030 and 2568, significant pathways were collapsed to nonredundant representatives, after which up to five pathways with positive NES and five pathways with negative NES were selected by increasing FDR and then decreasing absolute NES. Records of FGSEA were restricted to the Biological Process aspect and to taxon identifiers 5478 and 284593, and annotations carrying a NOT qualifier were removed. Protein accessions were mapped to gene identifiers from the DIA-NN annotation table. The gene set enrichment analysis was run with the proteins ranked by the limma moderated *t*-statistic. fgseaMultilevel [38] was run with pathway sizes of 10–500 mapped genes, eps = 0, scoreType = ‘std’, one processing thread, and a random seed of 1. Enrichment *p*-values were adjusted across tested pathways by the Benjamini–Hochberg procedure [36], and pathways with FDR < 0.05 were considered significant. The reported within-strain enrichment analysis was restricted to contrasts containing the strain-matched control. All six rankings were oriented as treatment minus control. Consequently, positive normalized enrichment scores consistently indicated treatment enrichment and negative scores indicated control enrichment. Redundancy among significant GO terms was reduced independently for every contrast with the function fgsea::collapsePathways [38]. For summary visualization, a maximum of five positive and five negative nonredundant pathways per contrast were selected by FDR.

Pathway-membership panels were aligned with the protein-abundance heatmap using ComplexHeatmap R package (version 2.28.0) [41].

### 4.8. Statistical Analysis

Fluorescence data obtained from the images, owing to their bounded nature (0–100%), were transformed using the arcsine square-root transformation [arcsin(√*p*)] prior to statistical analysis in order to stabilize variance. The number of biological replicates was *n* = 3. Because some groups in the transformed data did not meet the assumption of normality, as assessed with the Shapiro–Wilk test, differences between treatment groups were evaluated using the Kruskal–Wallis test followed by Dunn’s post hoc test. Multiple comparisons were accounted for by applying Holm’s correction, and the resulting *p*-values were adjusted accordingly. Results are presented as medians with interquartile ranges and displayed as box plots. For visual presentation, non-transformed data are shown. All statistical analyses were performed in Python 3.12 using SciPy (1.18.0), statsmodels (0.14.6), scikit-posthocs (0.14.0), pandas (3.0.4), numpy (2.5.1) and matplotlib (3.11.0).

## 5. Conclusions

This study provides new insight into the physiological and biochemical basis of the antibiofilm and antifungal activity of [(3-decanoylmethylamino)propyl]dimethylammonium acetate (AAB C9) and β-escin against *Candida glabrata*. By combining microscopic assessment of oxidative stress with quantitative proteomics in a reference strain ATCC 90030 and the clinical isolate 2586, we show that these compounds act primarily through membrane-mediated, pleiotropic mechanisms rather than through a single, well-defined molecular target.

Despite the expected interaction of both compounds with membrane lipids, gross cell morphology and membrane integrity were preserved under the sub-MIC_90_ conditions used here. Nonetheless, this apparently mild membrane perturbation was sufficient to trigger a measurable, strain-dependent increase in mitochondrial and superoxide-derived ROS: β-escin alone was the stronger oxidative stressor in the reference strain, whereas the AAB C9 + β-escin combination was most effective in the clinical isolate. This divergence points to differences in baseline stress tolerance or repair capacity between the two strains, with the clinical isolate apparently requiring combined exposure to reach a comparable level of oxidative challenge.

The comparison of the control proteomes demonstrated that strain background was the principal source of variation in the dataset, with the separation between ATCC 90030 and clinical isolate 2586 clearly exceeding the variation associated with treatment. On the other hand, the treatment comparisons demonstrated that AAB C9 and β-escin do not induce equivalent proteomic responses in ATCC 90030 and clinical isolate 2586. In the clinical isolate 2586, the principal response to C9 was associated with metabolic remodeling and a decrease in the abundance of proteins belonging to a biofilm-associated regulatory program, whereas β-escin exerted a broader influence on the proteome and largely dominated the response to the combined treatment. In ATCC 90030, C9 was associated with metabolic and membrane/cell-surface processes, β-escin with proteolysis and organelle organization, and the combination with transcriptional, RNA-related and intracellular trafficking responses.

Interestingly, treatment of *C. glabrata* strain 2586 with AAB C9 was associated with negative enrichment of nine biofilm forming-related proteins. The coordinated reduction in several regulators associated with the process suggests that AAB C9 may interfere with the regulatory program required to establish or maintain the biofilm.

Future work should determine whether these effects hold across a broader panel of clinical isolates, and whether longer exposure or higher sub-MIC concentrations amplify the strain-specific differences observed here. Moreover, a study with fungal biofilm instead of blastospores will help to clarify the potential of AAB C9 and β-escin as agents in antifungal and antibiofilm strategies.

## Figures and Tables

**Table 1:** Proteins associated with the GO term ‘biofilm formation’ and identified as downregulated upon treatment with AAB C9 in *C. glabrata* strain 2586. The table summarizes the UniProt accession number, respective protein name, and a short description.

Accession	Protein Name	Short Description
Q6FJB4	EFG1	HTH APSES-type domain-containing protein
Q6FJN8	SNF2	Transcription regulatory protein SNF2
Q6FKR3	HAA1	Transcription factor HAA1
Q6FM33	SIR4	Sir4 SID domain-containing protein
Q6FPB8	ZAP1	C2H2-type domain-containing protein
Q6FQL9	CST6	BZIP domain-containing protein
Q6FTZ3	MSS11	Transcription activator MSS11
Q6FX18	UPC2A	Zn(2)-C6 fungal-type domain-containing protein
Q6FXJ6	RIF1	Telomere-associated protein Rif1 N-terminal domain-containing protein

## Data Availability

The mass spectrometry proteomics data have been deposited to the PRIDE partner repository with the dataset identifier PXD083719.

## Supplementary Materials

The following supporting information can be downloaded at https://www.mdpi.com/article/10.3390/ijms27188309/s1.
